# The Construction and Analysis of lncRNA–miRNA–mRNA Competing Endogenous RNA Network of Schwann Cells in Diabetic Peripheral Neuropathy

**DOI:** 10.3389/fbioe.2020.00490

**Published:** 2020-05-25

**Authors:** Cheng Wang, Xiang Xu, Jing Chen, Yu Kang, Jiahe Guo, Dominik Duscher, Xiaofan Yang, Guojun Guo, Sen Ren, Hewei Xiong, Meng Yuan, Tao Jiang, Hans-Günther Machens, Zhenbing Chen, Yanhua Chen

**Affiliations:** ^1^Department of Hand Surgery, Union Hospital, Tongji Medical College, Huazhong University of Science and Technology, Wuhan, China; ^2^Department of Plastic and Hand Surgery, Technical University of Munich, Munich, Germany

**Keywords:** diabetic peripheral neuropathy, Schwann cells, mRNA, lncRNA, miRNA, RNA sequencing, competing endogenous RNA

## Abstract

**Background:**

Diabetes mellitus is a worldwide disease with high incidence. Diabetic peripheral neuropathy (DPN) is one of the most common but often ignored complications of diabetes mellitus that cause numbness and pain, even paralysis. Recent studies demonstrate that Schwann cells (SCs) in the peripheral nervous system play an essential role in the pathogenesis of DPN. Furthermore, various transcriptome analyses constructed by RNA-seq or microarray have provided a comprehensive understanding of molecular mechanisms and regulatory interaction networks involved in many diseases. However, the detailed mechanisms and competing endogenous RNA (ceRNA) network of SCs in DPN remain largely unknown.

**Methods:**

Whole-transcriptome sequencing technology was applied to systematically analyze the differentially expressed mRNAs, lncRNAs and miRNAs in SCs from DPN rats and control rats. Gene ontology (GO) and KEGG pathway enrichment analyses were used to investigate the potential functions of the differentially expressed genes. Following this, lncRNA-mRNA co-expression network and ceRNA regulatory network were constructed by bioinformatics analysis methods.

**Results:**

The results showed that 2925 mRNAs, 164 lncRNAs and 49 miRNAs were significantly differently expressed in SCs from DPN rats compared with control rats. 13 mRNAs, 7 lncRNAs and 7 miRNAs were validated by qRT-PCR and consistent with the RNA-seq data. Functional and pathway analyses revealed that many enriched biological processes of GO terms and pathways were highly correlated with the function of SCs and the pathogenesis of DPN. Furthermore, a global lncRNA–miRNA–mRNA ceRNA regulatory network in DPN model was constructed and miR-212-5p and the significantly correlated lncRNAs with high degree were identified as key mediators in the pathophysiological processes of SCs in DPN. These RNAs would contribute to the diagnosis and treatment of DPN.

**Conclusion:**

Our study has shown that differentially expressed RNAs have complex interactions among them. They also play critical roles in regulating functions of SCs involved in the pathogenesis of DPN. The novel competitive endogenous RNA network provides new insight for exploring the underlying molecular mechanism of DPN and further investigation may have clinical application value.

## Introduction

According to the International Diabetes Federation Diabetes Atlas, pre-diabetes and diabetes mellitus (DM) affect 425 million people worldwide in 2017, and this number will increase to 629 million in 2045 (International Diabetes Federation [IDF], 2017). Diabetic peripheral neuropathy (DPN) is one of most common chronic complications of both type 1 and type 2 diabetes mellitus, affecting at least half of patients diagnosed with diabetes. DPN is characterized by pain, tingling, paranesthesia, and hyperalgesia with a characteristic stocking–glove pattern. Previous studies indicated that hyperglycemia, insulin resistance, endothelial injury and microvascular dysfunctions play critical roles in the process of DPN (Gonçalves et al., 2017). However, the particular mechanisms in the pathogenesis of DPN remain poorly understood and the current gold standards of treatment is lifestyle intervention and rigorous glucose control.

Schwann cells (SCs), deriving from neural crest cells, are the most numerous glial cells of the peripheral nervous system (PNS) that form the myelin sheath around axons (Mizisin, 2014). SCs provide the important protective effect on axons by myelinating and ensheathing all axons in peripheral nerves, and have the potential to modulate neuronal biology. This is done by maintaining the endoneurial microenvironment via secreting various neurotrophic factors and the surrounding extracellular matrix (ECM), enriching the sodium channels, insulating the action potential velocity of axons, and promoting the regeneration of axons after nerve injury (Mizisin, 2014; Gonçalves et al., 2018). Previous investigations have demonstrated that peripheral nerve axonal demyelination and impaired remyelination in diabetic state are associated with Schwannopathy (Mizisin et al., 1998). Further studies conclusively have shown there are some key signaling pathways activated in SCs during diabetic neuropathy, such as hyperglycemia-driven high polyol pathway flux, oxidative stress, mitochondrial dysfunctions, dyslipidaemia and inflammation (Feldman et al., 2017; Gonçalves et al., 2018). However, the detailed mechanisms underlying changes of SCs in DPN remain to be investigated.

MicroRNAs (miRNAs) are a class of non-coding RNAs consisting of 20–22 nucleotides. They negatively regulate post-transcriptional expression levels of genes by binding to the 3′-untranslated region (UTR) to inhibit target mRNA translation or to degrade them (Bartel, 2004). Studies have increasingly found differentially expressed miRNAs play crucial roles in many human diseases, such as cancer, allergic diseases, metabolic diseases. It has been proved that miR-146a and miR-25 are involved in the pathogenesis of DPN as key regulators (Liu X. S. et al., 2017; Zhang et al., 2018). Long non-coding RNAs (lncRNAs) are a group of non-coding RNAs with the length of sequence more than 200 nucleotides. lncRNAs can interact with mRNAs and miRNAs to construct a complicated regulatory network by imposing an additional level of post-transcriptional regulation. Differentially expressed lncRNAs are linked to a variety of human diseases (Chen et al., 2013). Furthermore, increasing evidence has suggested that lncRNAs participate in the pathogenesis of DPN. For example, upregulation of lncRNA NONRATT021972 in dorsal root ganglia results in diabetic neuropathic pain of type 2 DM (Peng et al., 2017). Growing evidence supports that lncRNAs and circRNAs can act as competing endogenous RNAs (ceRNAs) or miRNA sponges which could competitively bind to miRNAs tends to regulate the expression levels of target mRNAs (Salmena et al., 2011; Zhang et al., 2019). However, the competing endogenous network of lncRNA–miRNA–mRNA of SCs in DPN still lacks systematic understanding and studying.

In our study, in order to obtain the expression profile of mRNAs, lncRNAs and miRNAs in diabetic SCs, DPN models were established. Then the RNA samples of SCs were collected from STZ-treated diabetic rats and control rats to conduct RNA-seq. All the expression levels of mRNAs, lncRNAs and miRNAs in SCs between two groups were screened and analyzed. Bioinformatics analyses, including Gene Ontology (GO) analysis, Kyoto Encyclopedia of Genes and Genomes (KEGG) pathway analysis, were performed to identify the significant biological functions and signaling pathways of DPN. 13 mRNAs, 7 lncRNAs and 7 miRNAs were confirmed as differentially expressed by qRT-PCR. Via these measures, ceRNA network construction was performed to reveal the regulatory mechanism of DPN and seek potential therapeutic targets.

## Materials and Methods

### Animals

Male Sprague-Dawley rats (*n* = 20, weighing 190–210 g) were obtained from Experimental Animal Center, Tongji Medical College, Huazhong University of Science and Technology, Wuhan, China. The animals were maintained under standard conditions of 12-h light/dark cycle and room temperature. Food and water were available *ad libitum*. The study was performed with the approval by the Animal Ethics Committee of Huazhong University of Science and Technology and all experimental procedures were carried out in accordance with the National Institutes of Health Guidelines and Regulations.

### Induction of Diabetes

Animals were randomly divided into two groups: diabetic group (*n* = 10) and control group (*n* = 10). After 12 h of fasting, rats of the diabetic group were treated with streptozotocin (STZ, Sigma-Aldrich, United States) injection at a dose of 65 mg/kg body weight dissolved in 0.05 mol/L citrate buffer, pH 4.5 at 4°C as described previously (Tong et al., 2012). The other group was treated with a single intra-peritoneal injection of an equal volume of citrate buffer without STZ as control. After STZ treatment for a week, the glucose meter (Accu-Chek Active; Roche, Germany) was used to monitor random blood glucose levels of all the rats from tail vein blood draws. Rats which exhibited blood glucose levels of 16.7 mM or higher were diagnosed with diabetes (Resham and Sharma, 2019) and enrolled in this study. Rats were kept to establish the model of DPN for 8 weeks (Yu et al., 2014). All animals survived until the end of the experiments.

### Behavioral Test

Behavior was tested by blinded observers. Mechanical allodynia was assessed by using von Frey filaments (Aesthesio, Danmic, United States) to stimulate plantar hind paws as described previously (Kroin et al., 2010; Santamaria et al., 2012). A 50% force withdrawal threshold was determined for the plantar hind paws using the “up-and-down” method (Chaplan et al., 1994). Tail-flick test and hot plate test were conducted to examine thermal hyperalgesia according to the techniques described previously (Sierra et al., 2015). Briefly, for the tail-flick test, rats were restrained in a fixation machine while tails were exposed. Approximately the distal 2/3 of the tail was immersed in the water bath maintained at 52.0 ± 0.2°C (Karna et al., 2019). The time was recorded when the tail removed or flicks. The cut off time for tail flick test was set at 10 s to avoid potential injury to the tail. For the hot plate test, a rat was placed into the Perspex cylinder on the hot-plate (Model 7280, Ugo Basile, Italy) of which the temperature was maintained 55.0 ± 0.2°C (Sierra et al., 2015). Response time for discomfort action or observed behavioral changes like licking paws, stomping or jumping was recorded. The cut off time for hot plate test was set at 15 s in order to avoid potential tissue injury. All the experiments were repeated three times.

### Electrophysiology

Sciatic nerve conduction velocity in two groups was tested as described previously (Ii et al., 2005; Hendrix et al., 2013; Baum et al., 2016). Briefly, both the motor nerve conduction velocity (MNCV) and sensory nerve conduction velocity (SNCV) were calculated through dividing the distance between the recording electrodes and the stimulation electrodes by the latency from the stimulation to the onset of the first peak signal (Ii et al., 2005). The MNCVs were recorded by the sterilized electrodes placed into the intrinsic foot muscles, and SNCVs were recorded by the near-nerve electrodes placed at the sciatic notch that were used as stimulation electrodes for MNCVs (Baum et al., 2016). During the experiments, the temperature of rats was kept at 37°C while the room temperature was maintained at 20 ± 0.5°C. The experiments were repeated three times and all data was collected and calculated by a Viking Quest Nicolet electromyography equipment and the specific software system semi automatically.

### Morphometric Analysis

After 8 weeks of STZ injection or vehicle solution injection and all the behavioral and electrophysiology test completed, the animals (*n* = 10 per group) were euthanized by using sodium pentobarbital and killed by cervical dislocation. Then all the sciatic nerves were dissected under sterile conditions. The mid-portion of right sciatic nerve (about 1–2 cm) from two groups (*n* = 3) was harvested sterilely. The parts of sciatic nerves were prefixed in 2.5% glutaraldehyde for 30 min and then post-fixed in 1% osmium tettroxide for 1 h, then embedded in epoxy resins. Ultra-thin (60 μm) sections were prepared and stained with uranium acetate-lead citrate. The transmission electron microscopy (HT7700, Hitachi, Japan) was used to image the samples of sciatic nerves. For each sample at least three pictures were taken to assess the development or progression of DPN (Yang et al., 2018). Subsequently, the diameter of myelinated nerve fibers and the mean of g-ratio (the ratio of the inner axon diameter to the outer myelinated fiber diameter) were calculated by using Image J software for further statistical analysis.

### Isolation of Primary Schwann Cells and Culture Preparations

The left sciatic nerve (between sciatic notch and the ankle) and the remaining segment of the right sciatic nerve were isolated from each rat sterilely. After stripping off the whole epineurium by using microsurgical instruments under the stereomicroscope, nerves were cut into segments of 1cm length. The segments were cultured in pre-degeneration medium containing SCM (Schwann Cell Medium, ScienCell, United States), 10% FBS (fetal bovine serum, ScienCell) and 1% penicillin–streptomycin for 10 days at 37°C and 5% CO^2^. After pre-degeneration, the peripheral nerve tissue was cut into short segments of 2–4 mm length and dissociated enzymatically for 20 h at 37°C and 5% CO^2^. Dissociation medium consisted of DMEM (Thermo, United States), 10% FBS, 0.125% collagenase type IV (Sigma-Aldrich), 1.25 U/ml dispase II (Solaribo, China) and 1% penicillin–streptomycin (Haastert et al., 2007). Then cell suspensions were centrifuged at 235 *g* for 5 min at +21°C, rinsed in DMEM, centrifuged again and resuspended in SC culture medium containing SCM, 5% FBS, 1% SCGS (Schwann Cell Growth Supplement, ScienCell) and 1% penicillin–streptomycin. Then the cell suspensions were plated at 106 cells per well on a poly-L-ornithin (Sigma-Aldrich) and laminin (Sigma-Aldrich) coated six well plate. The medium was changed to serum-free SC culture medium after 24 h. SCs were enriched and purified as described previously (Haastert et al., 2007; Kaewkhaw et al., 2012). Specially, SCs dissociated from control groups and STZ-treated groups were cultured in SC culture medium supplemented with 5.6 mM glucose (for control groups), 30 mM glucose (for STZ-treated groups) (Walsh et al., 2013).

### Immunofluorescent Staining

The purity of SCs was determined by immunostaining. After being fixed in 4% paraformaldehyde and permeabilized with 0.2% Triton X-100 (Sigma-Aldrich), SCs were incubated with primary antibody S-100β (1: 100, ProteinTech Group, Inc., United States) at 4°C overnight and then incubated with the cy3 conjugated goat anti-rabbit IgG secondary antibody (1: 200, ProteinTech) for 1 h in the darkness. After being washed with PBS (phosphate-buffered saline, pH 7.4), the cells were incubated with 4′,6-diamidino-2-phenylindole (DAPI, Boster Biological Technology, China) to label nuclei. The purity of SCs was obtained based on the proportion of S-100β-positive cells with DAPI-stained nuclei.

### RNA Extraction and RNA-Seq Analysis

Schwann cell samples were randomly selected from control groups (*n* = 3) and diabetic groups (*n* = 3), rinsed with PBS twice and total RNA was isolated by using miRNeasy Mini Kit (Qiagen, Germany) according to the manufacturer’s instructions. The concentration and quality of RNA samples were assessed by the NanoPhotometer spectrophotometer (IMPLEN, USA). All RNA samples had an RNA Integrity Number (RIN) > 9.0 (Supplementary File S1). After total RNA from each sample was qualified, RNA-seq was assessed to identify the differentially expressed mRNAs, lncRNAs and miRNAs in the DPN rat models. Illumina TruSeq RNA Sample Prep Kit was used with 1 μg of total RNA for the construction of sequencing libraries on the Illumina HiSeq-Xten sequencing platform (Genminix Informatics Ltd, Shanghai, China). Raw reads were filtered and cleaned using fastp version 0.19.4 to remove the adaptor reads and low-quality tags (Yang et al., 2018). Then the sequence reads were aligned to the reference genome using HISAT2 for transcripts and Bowtie v.1.2.2 for miRNA as described (Langmead, 2010; Kim et al., 2015). Subsequently, raw counts were generated using the Stringtie v1.3.4 tool for transcripts and the featureCounts v1.6.1 tool for miRNA (Liao et al., 2014; Pertea et al., 2015). Differential expression analysis was performed using DESeq2 v1.20.0 (Love et al., 2014).

### Cell RNA Extraction and Quantitative Real-Time PCR (qRT-PCR)

The remaining SCs samples in each group (*n* = 5 per group) were applied to validate RNA-seq results by using quantitative real-time polymerase chain reaction. Total cell RNA was extracted with TRIzol Reagent (Invitrogen, United States). For miRNA, 2 μg of total RNA was reverse transcribed to cDNA using the Mir-X miRNA First-Strand Synthesis kit (Clontech, Japan) according to the user manual. For mRNA and lncRNA, 1 μg of RNA was reverse transcribed using Takara reverse transcription kit (Takara, China) and qRT-PCR was performed based on the instruction of Mir-X miRNA qRT-PCR SYBR Kit (Clontech) and ChamQ SYBR qPCR Master Mix (Vazyme, China), respectively. The delta–delta Ct method (2^–ΔΔCt^) was used to measure the relative levels of transcripts expression and the data were normalized to β-actin (endogenous internal controls of mRNA and lncRNA) or U6 (endogenous internal control of miRNA). The experiment was repeated three times. The primer sequences (Supplementary Table S1) were synthesized by TSINGKE Biological Technology Co., Ltd (China).

### Functional and Pathway Enrichment

Gene Ontology (GO) analysis and Kyoto Encyclopedia of Genes and Genomes (KEGG) pathway analysis were performed to investigate biological functions of DPN-related genes. The ontology contains three domains: biological process, cellular components, and molecular interaction^1^ and KEGG^2^ is a collection of online databases. Briefly, GO analysis was applied to annotated function of differentially expressed genes in SCs based on GO database. After analysis for the significance level (*P*-value) and false discovery rate (FDR), GO terms were selected from the enriched gene sets (FDR < 0.05). Pathway analysis was performed to explore pathway clusters covering the molecular interaction and reaction networks of the differentially expressed genes according to KEGG. *P*-value < 0.05 was considered as significant pathways.

### Construction of lncRNA–mRNA Network and Prediction of miRNA Targets

Based on the theory of weighed gene co-expression network analysis (WGCNA) (Zhang and Horvath, 2005; Horvath, 2011), the method of lncRNA-mRNA co-expression network construction was consistent with previous study (Jiang et al., 2016). The co-expression similarity S_ij_ is defined and calculated as the absolute value of the Pearson correlation coefficient between the expression profiles of two genes i and j:

(1) $${\text{S}}_{\text{ij}}=\left|c\,o\,r\,\left(i,j\right)\right|$$

Then S_ij_ is transformed into the adjacency a_ij_ via an adjacency function:

$${\text{a}}_{\text{ij}}={\text{S}}_{\text{ij}}^{\beta }$$

In this formula, the positive integer β represents the exponential parameter for power law distribution and is used to characterize the likeness to a scale-free network (Langfelder and Horvath, 2008). The connectivity of the i-th node is defined by

$${\text{k}}_{\text{i}}=\sum_{\text{j}}{\text{a}}_{\text{ij}}$$

In unweighted networks, the connectivity k_i_ equals the number of nodes that are directly linked to node i. In weighted networks, the connectivity equals the sum of connection weights between node i and the other nodes. And the distribution of connectivity (k) of genes best fits a power law p(k) ∼ k^–γ^ (Barabási, 2009). With 1∼30 of β value ranges, the linear model was established by logarithms of the adjacency degree of a node log(k) and the appearance probability of this node log(p(k)) and ensuring that the overall average connectivity was not too low (Zhao and Fan, 2019). β = 22 was finally determined for further analysis according to scale free topology criterion and p(k) in our co-expressing network indicates the probability that a gene is co-expressed with k other genes. Next, the dissimilarity between the nodes can be calculated by the topological overlap measure (TOM) and was used as the distance. The hybrid hierarchical clustering algorithm was used to perform cluster analysis in order to identify gene modules for biological significance. The module reflects the subset of tightly connected nodes in the network. Furthermore, genes in high modules may be important candidate hub genes which have important biological functions (Ravasz, 2002; Yip and Horvath, 2006; Lim et al., 2013). The lncRNA-mRNA network was drawn by Cytoscape 3.7.2^3^. The targets of differentially expressed miRNAs were predicted by miRanda and annotated with miRTarBase (Joung et al., 2007). The predicted targets involved mRNAs and lncRNAs were compared to differentially expressed mRNAs and lncRNAs detected from RNA-Seq, and the overlapped RNAs were collected. Then, the differentially expressed miRNAs and selected target mRNAs and lncRNAs were combined for RNA network analyses.

### Construction of the ceRNA Regulatory Work

**FIGURE 1:**
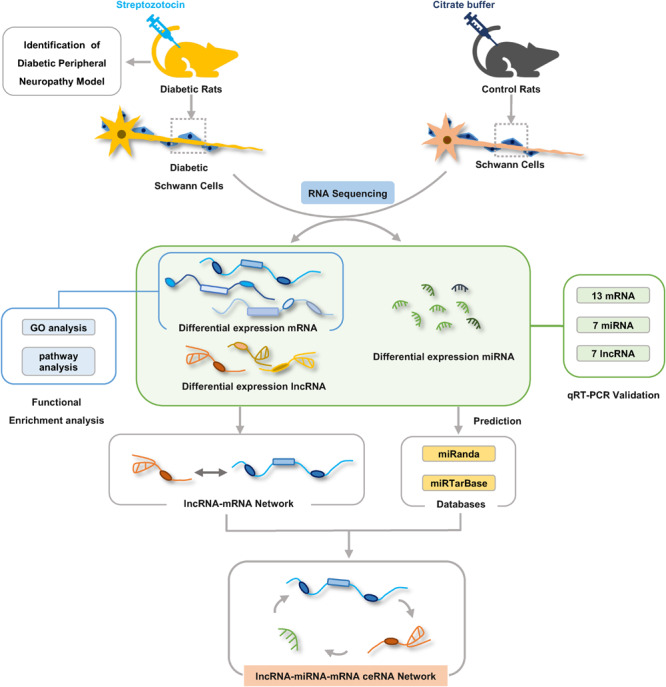
The workflow of experiments and bioinformatic analysis. Male Sprague-Dawley rats were treated with streptozotocin (STZ) or citrate buffer and after 8 weeks Schwann cells were isolated from sciatic nerves of rats for RNA-seq analysis. Gene ontology and KEGG pathway enrichment analyses were used to investigate the potential functions of the differentially expressed genes. lncRNA–mRNA co-expression network and ceRNA regulatory network were constructed by bioinformatics analysis methods.

Some RNA molecules such as lncRNAs and mRNAs are able to communicate with each other by sharing miRNA response elements (MREs) according to the ceRNA hypothesis (Salmena et al., 2011). To investigate the role and interaction among mRNAs, lncRNAs, and miRNAs in DPN pathogenesis, we constructed the ceRNA network based on miRNA-target relationship and co-expressed relationship between mRNA and lncRNA. The network was constructed in four steps in detail: (1) Obtention of the differential expressed mRNAs, miRNAs and lncRNAs by RNA-seq; (2) Prediction of the targeting relationship between differential miRNAs and differential mRNAs (tool: miRanda). miRanda predicted the targeting relationship between miRNA and mRNA based on the sequence alignment and binding free energy calculation of miRNA sequences and 3′-UTR of mRNA. (3) Prediction of the targeting relationship between differential miRNAs and differential lncRNAs (tool: miRanda); (4) Construction of ceRNA network: differentially expressed lncRNA-mRNA pairs were identified based on Pearson correlation coefficients (PCC) calculated from differentially expressed mRNAs and lncRNAs that were identified in our RNA-seq results (PCC > 0.8 and *p* < 0.05). Then we used the targeting relationship of mRNA/lncRNA regulated by microRNA to establish a lncRNA–miRNA–mRNA interaction network. Based on the targeting relationship between mRNA/lncRNA and microRNA, the adjacency matrix A = [a_ij_] of mRNA/lncRNA and microRNA could be constructed, a_ij_ represents the weight of the relationship between mRNA/lncRNA i and microRNA j. Finally, the ceRNA network was constructed using Cytoscape v3.7.2 according to predicted shared pairs of miRNA–mRNA and miRNA–lncRNA and the differentially expressed lncRNA-mRNA pairs (Otasek et al., 2019). The significance of shared miRNAs in this primary network was further calculated using the hypergeometric test (*p* < 0.05) (Le et al., 2017). The importance of RNA molecules in the network is expressed by the degree of the network. The degree of centrality indicates the degree of contribution of microRNA to surrounding mRNA/lncRNA, or the degree of contribution of mRNA/lncRNA to surrounding microRNA. The complete experiment workflow is shown in Figure 1.

### Dual-Luciferase Reporter Assay

To validate whether miR-212-5p directly targets the Gucy1a3 3′-UTR, dual-luciferase reporter assay was performed as described previously (Yu et al., 2012). In brief, the wildtype (WT) and mutated (MUT) 3′-UTR regions of Gucy1a3 were cloned and inserted into the *Mlu*I and *Hin*dIII restriction enzyme sites of the pMIR-Report vector (Obio Technology Co., Ltd, Shanghai, China). HEK-293T cells were seeded in 96-well plates for 24 h. Then, Gucy1a3-WT, Gucy1a3-MUT reporter or pMIR empty vector plasmid was co-transfected with Renilla luciferase (pRL) plasmids and miR-212-5p mimic or negative control (NC) using lipofectamine 2000 (Invitrogen). Forty eight hours after transfection, the Dual Luciferase Reporter Assay System (Promega, United States) was used to analyzed luciferase activity. The experiments were repeated three times.

### Statistical Analysis

Data were expressed as the mean ± SEM. The unpaired Student’s *t*-test and one-way analysis of variance with Bonferroni *post hoc* test were performed for comparisons between two groups. *P* < 0.05 was considered statistically significant. Pearson correlation analysis was used to assess the ceRNA network construction. Statistical analysis was calculated with the GraphPad Prism v 8.1 software.

## Results

### Model Identification of Diabetic Peripheral Neuropathy

**FIGURE 2:**
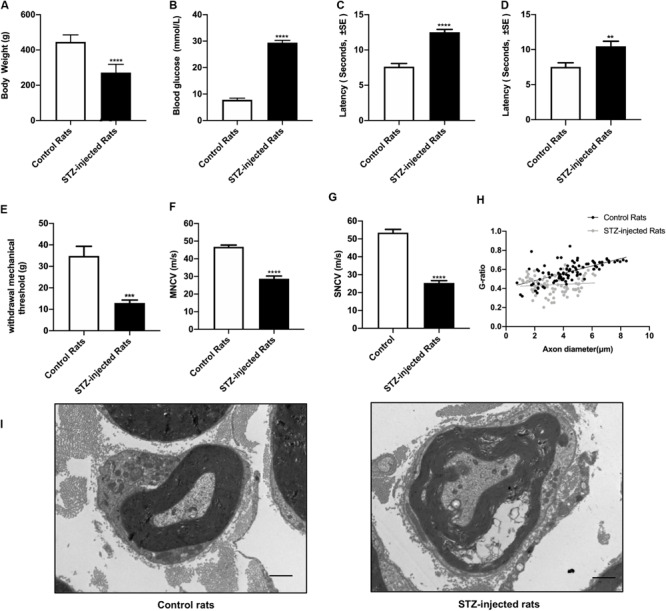
Evaluation of streptozotocin (STZ)-injected diabetic peripheral neuropathy (DPN) rat models. The differences between streptozotocin (STZ)-treated rats and control rats were calculated. The non-fasting blood glucose levels **(B)** were significantly higher in STZ-injected rats compared with control rats. The body weight **(A)**, withdrawal threshold **(E)** and the motor and sensory nerve conduction velocities **(F,G)** in STZ-injected rats significantly decreased. The latency of hot plate test **(C)**, tail flick test **(D),** and g-ratio of myelinated nerve fibers **(H)** significantly increased compared with controls (mean ± SEM, *n* = 10 per group, *p < 0.05, **p < 0.01, ***p < 0.001, ****p < 0.0001). The detail of myelinated fibers was showed in **(I)**. Scale bars: 1.0 μm.

Eight weeks after STZ injection, the body weight, the blood glucose levels, the threshold of thermal and mechanical stimuli, nervous conduction velocity and the morphological changes of sciatic nerve were detected to assess the signs and symptoms of DPN. The results showed that compared with control groups, the increased non-fasting blood glucose levels of all the rats in STZ-treated group were in accordance with diagnostic standard of diabetes (Figure 2A), whereas the body weight decreased (Figure 2B), indicating the diabetic rat models were established successfully. The sensory functions of sciatic nerve were examined by tail flick test, hot plate test, and von Frey hair test. Furthermore, both the motor and sensory nerve conduction velocity of sciatic nerve were investigated. In agreement with previous studies, the slowing down of nerve conduction (Figures 2F,G), the increase of the mechanical threshold (Figure 2E) and decrease of thermal threshold (Figures 2C,D) revealed the damage of sciatic nerve caused by DPN (Mitro et al., 2014; Sierra et al., 2015; Watson and Dyck, 2015). Morphological analysis showed the g-ratio of myelinated fibers were significantly affected by DPN (Figure 2H). In addition, nerve fibers from diabetic rats showed swollen fibers and the loss of myelinated fibers (Figure 2I).

### Characterization of SCs

**FIGURE 3:**
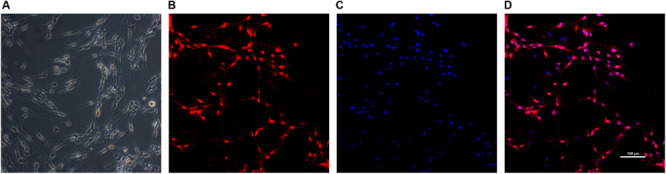
Identification of SCs. The morphology of Schwann cells **(A)** was observed under light microscope. SCs were immunolabeled for S100β **(B)** and nucleic acid was signed with DAPI **(C)**. Image **(D)** was merged from **(B,C)**. Scale bar: 100 μm.

Schwann cells at passage 2 were typically bipolar, tripolar or multipolar morphology *in vitro* while fibroblasts were more flattened polygonal contour than SCs under light microscope (Kaewkhaw et al., 2012) (Figure 3). The immunofluorescent staining results showed the purity of SCs reached 87.0 ± 4.9% for S-100β antibody marker.

### Differentially Expressed lncRNAs, miRNAs, mRNAs in Schwann Cells of Rats With DPN

**FIGURE 4:**
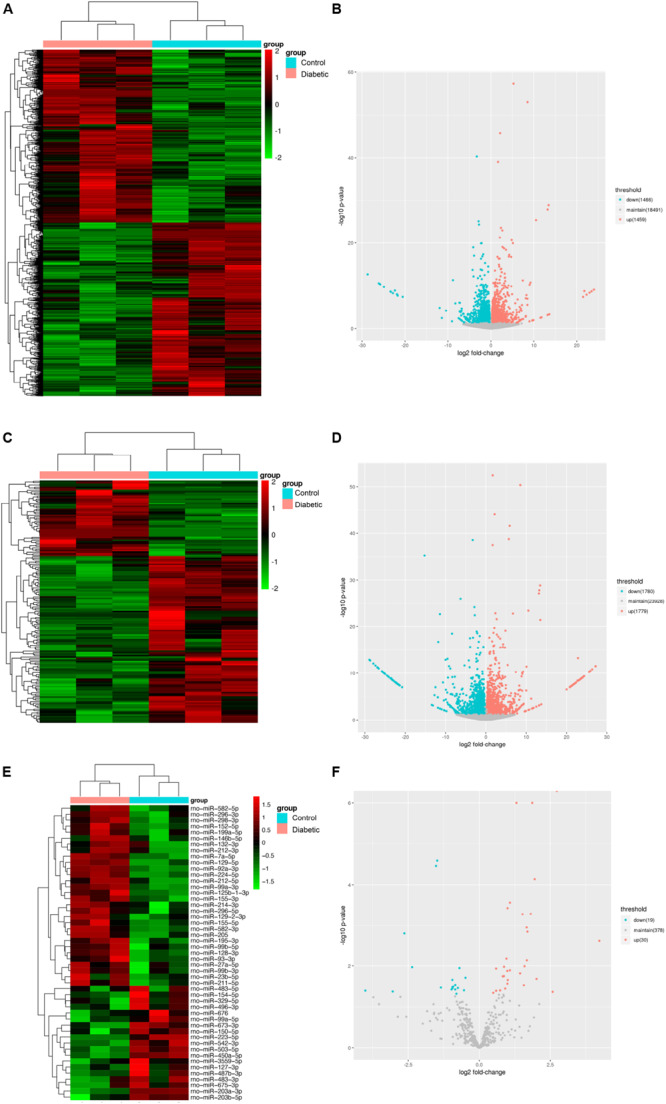
Identification of mRNAs, lncRNAs, and miRNAs differentially expression in Schwann cells from streptozotocin (STZ)-treated rats and control rats. Hierarchical clustering analysis showed all differentially expressed mRNAs **(A)**, lncRNAs **(C),** and miRNAs **(E)**. Red color represented relatively high expression and green color represents relatively low expression. Volcano plots of normalized expression levels for differentially expressed mRNAs **(B)**, lncRNAs **(D)** and miRNAs **(F)**. The red points in the plots represented the upregulated RNAs with statistical significance and the blue points in the plots represented the downregulated RNAs with statistical significance (*P*-value < 0.05 and fold change > 1.2 or <0.83).

To identify functional mRNAs, lncRNAs, and miRNAs involved in SCs of rats with DPN, total RNA of SCs randomly selected from three diabetic groups and three control groups were collected for RNA-Seq. 4,622 lncRNAs, 427 miRNAs and 32,883 mRNAs were detected in total. The results showed that 164 lncRNAs, 49 miRNAs and 2,925 mRNAs were significantly altered with fold changes > 1.2 or <0.83 in SCs of diabetic groups (Bonfanti et al., 2015) (Supplementary Tables S2–S4, *P*-value < 0.05). All the differentially expressed lncRNAs, miRNAs, and mRNAs were presented by the hierarchical clustering heat maps and volcano plots in Figure 4. Specifically, among all the differentially expressed lncRNAs, 51 lncRNAs were significantly upregulated whereas 113 lncRNAs were significantly downregulated in diabetic groups. Thirty miRNAs were significantly increased and 19 miRNAs were significantly decreased. Among the differentially expressed mRNAs, a total of 1,459 mRNAs were upregulated and 1,466 mRNAs were downregulated in the diabetic groups compared with control groups.

### Validation of Differentially Expressed mRNAs, lncRNAs, and miRNAs in Schwann Cells of DPN

**FIGURE 5:**
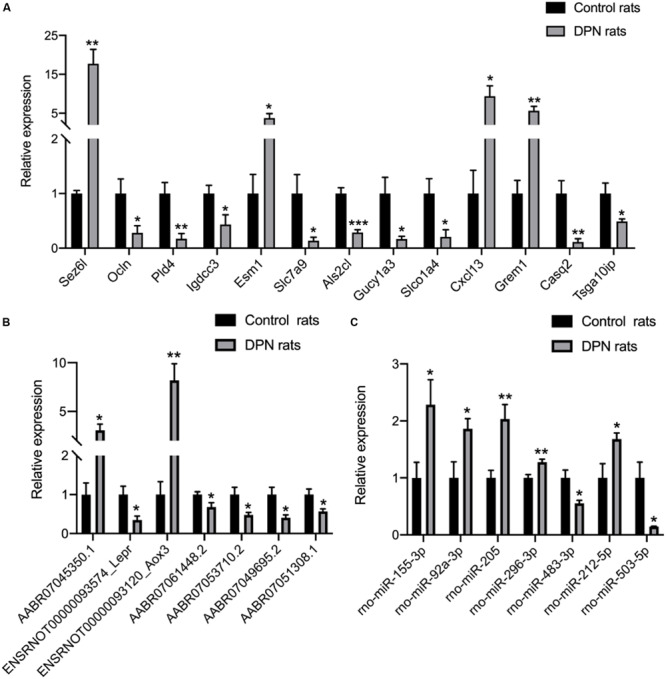
Confirmation of RNA-seq data in Schwann cells from streptozotocin (STZ)-treated rats and control rats by qRT-PCR. Thirteen mRNAs **(A)** were consistent with the RNA-seq results. Seven lncRNAs **(B)** were consistent with the RNA-seq results. Seven miRNAs **(C)** were consistent with the RNA-seq results. Results were presented as mean ± SEM of five independent experiments (*p < 0.05, **p < 0.01, ***p < 0.001, ****p < 0.0001). RNA samples used for qRT-PCR analysis were independent of RNA-seq samples.

qRT-PCR analysis was performed on RNA extracted from SCs to confirm the expression level changes gained from the RNA-seq analysis. We randomly selected 21 mRNAs, 13 mRNAs and 9 miRNAs with high fold change to validate the results of RNA-seq. 13 mRNAs, 7 lncRNAs and 7 miRNAs had been verified. The relative expression levels of the validated mRNAs, lncRNAs and miRNAs were showed in Figures 5A–C, respectively. The qRT-PCR results were found to be consistent with the RNA-seq data.

### Functional Enrichment Analyses

**FIGURE 6:**
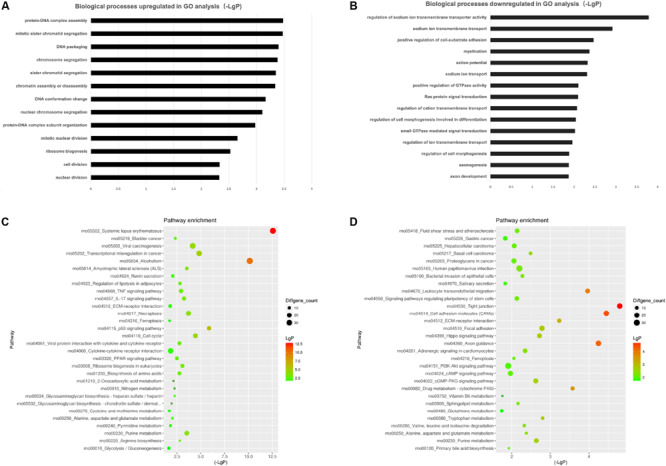
Gene ontology (GO) analysis and KEGG pathway analysis. GO annotation of biological processes (BP) related to upregulated and downregulated mRNAs **(A,B)** of DPN model. KEGG pathway enrichment analysis of upregulated and downregulated mRNAs **(C,D)** of DPN model.

To investigate the biological effects of mRNAs, lncRNAs and miRNAs in SCs of DPN rats, GO analysis and KEGG pathway analysis were performed. GO analysis was used to analyze the up and down-regulated differentially expressed targeted genes individually. The GO biological process analysis showed that 6,258 downregulated biological process of GO terms and 6,321 upregulated biological process of GO terms were enriched. Of which, 147 GO terms were significantly downregulated and 320 GO terms were significantly upregulated (Supplementary Table S5, FDR < 0.05). A fraction of significant enriched terms was listed in Figures 6A,B. Some significant GO terms were associated with the changes of SCs’ function such as myelination, action potential, axongenesis and axon development (Figure 6A). Pathway enrichment analyses were performed to explore crucial signaling pathways of DPN. The results showed 36 upregulated and 46 downregulated remarkable enriched pathways in Supplementary Table S6. The top downregulated signaling pathways included many pathways related to SCs, such as cell adhesion molecules (CAMs), ECM-receptor interaction, axon guidance, and others related to intracellular pathways, such as PI3K-Akt signaling pathway, cAMP signaling pathway (Figures 6C,D). Among the top upregulated signaling pathway, immune response pathway and metabolism pathway were involved like IL-17 signaling pathways, TNF signaling pathway, p53 signaling pathway and PPAR pathway.

### lncRNA–mRNA Network Construction and miRNA Target Prediction

**FIGURE 7:**
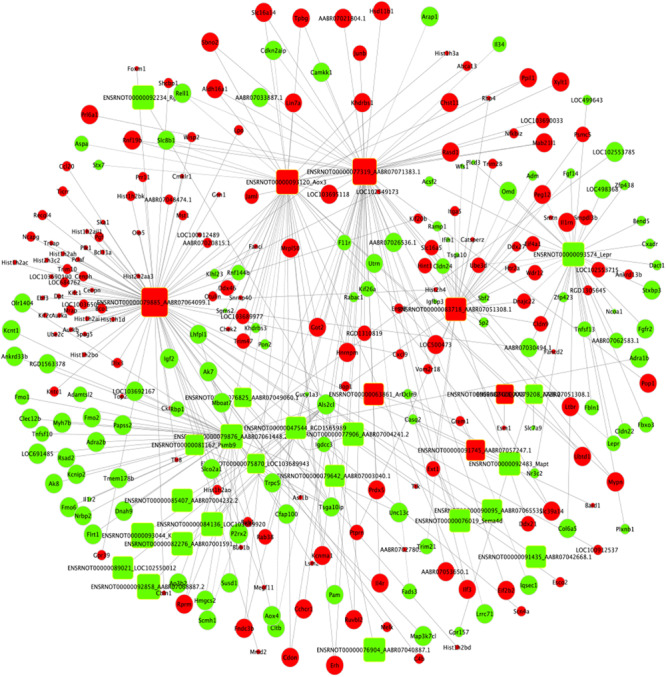
Sub-lncRNA–mRNA regulatory network was established based on 147 mRNAs and 9 lncRNAs. lncRNA and mRNA were indicated to round rectangle and ellipse, respectively. The red nodes represented upregulated RNAs and green nodes represented downregulated RNAs. The size of a node indicated the degree of node in the network.

Based on the expression correlation between the differentially expressed mRNAs and lncRNAs, the lncRNA-mRNA co-expression network was constructed firstly. The whole network consists of 161 lncRNAs and 1,511 mRNAs (Supplementary Table S7 and Supplementary Figure S1). Among the network, the lncRNA ENSRNOT00000079885_AABR07064099.1 had the highest degree of 78 and the mRNAs Kcnt1, LOC691485 and P2rx2 had the highest degree of 13. Considering the figure cannot clearly display the complex relationship in whole network, nine top lncRNAs (degree ≥ 45) and related 147 mRNAs were selected to construct sub-lncRNA-mRNA network (Figure 7). Moreover, miRanda and miRTarBase database were used to predict the target mRNAs and lncRNAs (Supplementary Table S8). As the results, 2,634 mRNAs and 178 lncRNAs were predicted from 49 differentially expressed miRNAs identified from RNA-seq.

### Construction of ceRNA Network

**FIGURE 8:**
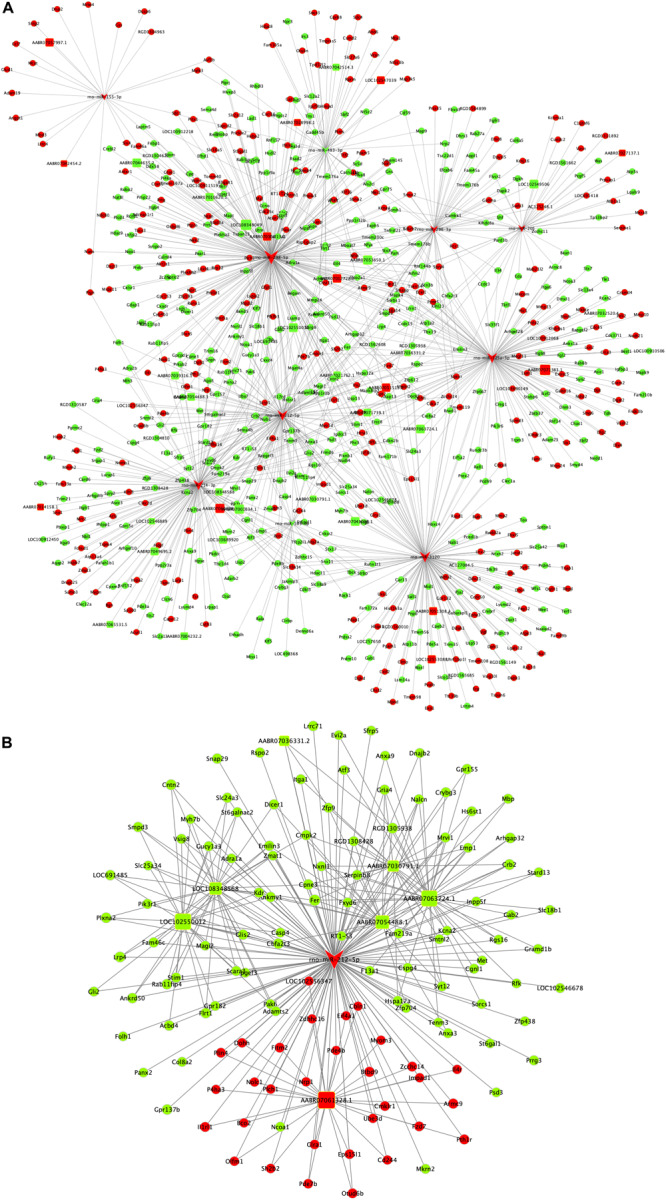
Competing endogenous RNA network in DPN model. The competing endogenous RNA network is based on miRNA-target relationship and co-expressed interactions between mRNA and lncRNA. The whole competing endogenous RNA network **(A)** in the DPN model. The sub competing endogenous RNA network **(B)** consists of miR-212-5p, 7 lncRNAs and 125 mRNAs. miRNA was indicated to V-shape, lncRNAs were indicated to round rectangle and mRNAs were indicated to ellipse. The red nodes represented upregulated RNAs and green nodes represented downregulated RNAs. The size of a node indicated the degree of node in the network.

**FIGURE 9:**
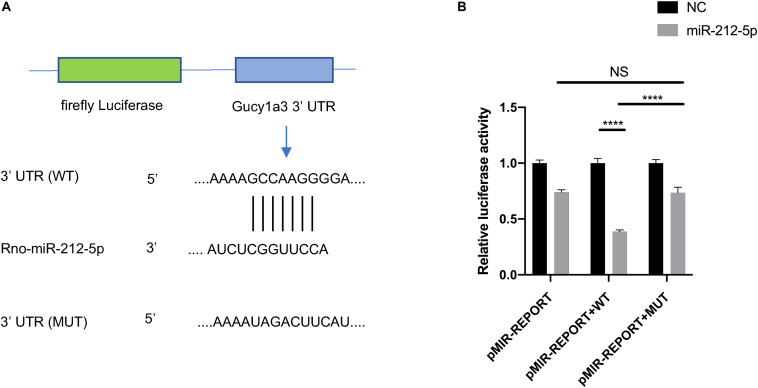
The interaction between Gucy1a3 and miR-212-5p. Potential binding sites of miR-212-5p and 3′ UTR of Gucy1a3 mRNA predicted by TargetScan **(A)**. Dual-Luciferase activity assay showed that miR-212-5p could bind with the 3′ UTR of Gucy1a3 mRNA **(B)**. (*p < 0.05, **p < 0.01, ***p < 0.001, ****p < 0.0001).

The ceRNA regulatory network was established in order to reveal the potential molecular mechanism in SCs involved in DPN pathogenesis based on the ceRNA hypothesis. In this study, 53 miRNAs, 59 lncRNAs and 1,229 mRNAs were found by bounding the number of miRNA response elements (MREs) and the number of miRNAs shared by lncRNA-mRNA more than 1 (Supplementary Table S9 and Supplementary Figure S2). Considering the figure cannot clearly display the complex relationships among 1,341 RNAs that constituted the ceRNA network, we selected miRNAs which had higher degree in the network and validated by qRT-PCR to construct the network diagram involving 38 lncRNAs, 10 miRNAs, and 702 mRNAs (Figure 8A). Based on the validation results of qRT-PCR and the connection with diabetes, miR-212-5p and predicted target Gucy1a3 was selected to further display the ceRNA network (Figure 8B). The lncRNAs such as LOC108348568, AABR07030791.1, LOC102550012, AABR07054488.1, AABR07036331.2, AABR07061328.1, AABR07063724.1, and AABR07063724.1 associated with miR-212-5p was also showed in Figure 8B. To further confirmation, dual-luciferase reporter assay was used to verify the relationship between miR-212-5p and Gucy1a3. The result showed that miR-212-5p could target the Gucy1a3 3′-UTR and decreased luciferase activity (Figure 9), which was consistent with the results of RNA-seq and qRT-PCR. However, the luciferase activities in the mutant group and empty control group with miR-212-5p mimics also decreased (Figure 9B). We analyzed that the main reason of this result was the potential interaction between the pMIR-REPORT Luciferase vector and miR-212-5p mimics.

## Discussion

Diabetes mellitus is a worldwide disease with high incidence, and a growing number of DM patients are suffering from DPN (Feldman et al., 2017). A large amount of review and analysis of the literature suggest the possible mechanism in DPN development (Mizisin, 2014; Gonçalves et al., 2017, 2018). Nevertheless, there is a lack of the effectual treatment for diabetic neuropathy and neuropathic pain. SCs play a vital role in the process of DPN (Gonçalves et al., 2017). Recent experimental data provide further insight into the complex relationship between dysfunctional SCs and neuropathy. To better explore the new targets for early diagnosis and treatment, the specific molecular mechanism of SCs in DPN need to be understood. In this study, 8 weeks after streptozotocin injection, the DPN model in rats was established (Zochodne et al., 2007). Moreover, adult rat SCs were isolated and cultured successfully. In order to elucidate the detailed mechanism and the pathogenesis of DPN, RNA-seq technology was applied to systematically analyze the differentially expressed lncRNAs, miRNAs and mRNAs of SCs from DPN rats and the ceRNA regulatory network was established.

In the present study, 2,925 mRNAs, 164 lncRNAs and 49 miRNAs were significantly upregulated or downregulated in SCs of DPN rats. The expression levels of mRNAs, lncRNAs, and miRNAs were confirmed in the model of DPN via qRT-PCR validation and the results were in accordance with RNA-seq data. Furthermore, some confirmed RNAs have been reported associated with diabetes mellitus or DPN in the past few years. For example, chemokine CXCL13 (C-X-C motif chemokine ligand 13) and its receptor CXCR5 (C-X-C chemokine receptor type 5) associated with inflammatory response have been demonstrated directly to be a pivotal regulator in the pathogenesis of painful diabetic neuropathy via ERK pathway (Liu et al., 2019). The study shows that Ocln (occludin), a tight junction protein involved in myelin sheath related to SCs, has significant differences in patients with demyelinating peripheral neuropathies (Manole et al., 2015). Gene Grem1 and Slc7a9 are associated with diabetic nephropathy (McKnight et al., 2010; Guan et al., 2016). It is reported that miR-483-3p could target vascular endothelial zinc finger 1 (VEZF1) to modulate endothelial integrity in patients with type 2 diabetes (Kuschnerus et al., 2019). Studies also show that miR-212-5p regulated lipid metabolism targeting fatty acid synthase (FAS) and stearoyl-CoA desaturase-1 (SCD1) (Guo et al., 2017). In addition, miRNA-212-5p is related to the neuron death in the central nervous system (Sun et al., 2018; Xiao et al., 2019). Moreover, recent studies have also reported the integral roles of lncRNAs in DPN (Liu C. et al., 2017; Peng et al., 2017), and previous studies apply microarray technology to analyze the differentially expressed lncRNAs in dorsal root ganglia from STZ-induced diabetic rats (Du et al., 2019; Guo et al., 2019). These differentially expressed RNAs may be a potential target for diagnosis and therapy of DPN.

To further investigate in the potential biological implications of changes in RNAs of SCs in DPN, GO analysis and KEGG pathway analysis were carried out. In biological process fold enrichment of GO analysis, a mass of significant enriched downregulated GO terms such as axon development (GO:0061564), axonogenesis (GO:0007409), action potential (GO:0001508), myelination (GO:0042552), and axon ensheathment (GO:0008366) showed that diabetes contributed to dysfunction of SCs and nerve degeneration which consistent with previous studies (Sango et al., 2006; Mizisin, 2014). In addition, there is obvious co-relevance between Schwannopathy and DPN. The causes for the changes in structure and function of peripheral nerve are the hyperglycemia-driven SC stress and neurodegeneration that leads to DPN (Gonçalves et al., 2017).

According to the pathway analyses, 36 upregulated and 46 downregulated enriched pathways were obtained to further explain the underlying functions of differentially expressed RNAs. The remarkable downregulated pathways involved tight junction (rno04530), cell adhesion molecules (CAMs, rno04514), axon guidance (rno04360) and PI3K-Akt signaling pathway (rno04151) while the upregulated pathways involved p53 signaling pathway (rno04115), TNF signaling pathway (rno04668), PPAR signaling pathway (rno03320) and AGE-RAGE signaling pathway in diabetic complications (rno04933). Interestingly, there are many signal conduction paths and molecular mechanisms that may participate in the pathological progression of DPN which has been reported. AGE-RAGE signaling pathway is a significantly enriched pathway that plays roles in regulating the sustained oxidative stress and inflammation that occurs in DPN (Wang et al., 2019). Importantly, hyperglycemia-induced excess formation of advanced glycation end product (AGE) results in axonal demyelination and degeneration, the structure and function changes of SCs and the detrimental effects on axons (Yagihashi et al., 1992; Sugimoto et al., 2008). In addition, the receptor for AGE (RAGE) is expressed in endothelial and SCs and the interactions between AGEs and RAGE induce to the Schwannopathy and microangiopathy in the peripheral nerve under diabetic state (Wada and Yagihashi, 2005). Furthermore, gentiopicroside has been demonstrated to have protective effect on DPN through PPAR-γ/AMPK/ACC signal pathway (Lu et al., 2018). The neuroprotective effects of Heparin-Poloxamer thermosensitive hydrogel co-delivered with basic fibroblast growth factor (bFGF) and nerve growth factor (NGF) on diabetic rats are mainly attributed to the activation of phosphatidylinositol 3 kinase and protein kinase B (PI3K-Akt) signaling pathway and mitogen-activated protein kinase kinase/extracellular signal-regulated kinase (MAPK/ERK) signaling pathway (Li et al., 2018). Moreover, a vast array of signaling pathways involving axon guidance, TNF-α signaling, loss of tight junction which accounted for the pathogenesis of DPN, have been mentioned in many literatures (Pande et al., 2011; Dewanjee et al., 2018). In this study, the enrichment of the above-mentioned pathways suggests the possible therapeutic targets of DPN.

The most possible ceRNA network involved in the pathogenesis and development of DPN was selected and constructed. Rno-miR-212-5p had the high degree in the whole ceRNA network while its predicted targets (according to the target prediction database, miRanda) included Gucy1a3 (encoding guanylate cyclase soluble subunit alpha-3). Both of them were listed as differentially expressed RNAs and validated by qRT-PCR. Gucy1a3, a subunit of soluble Guanylyl Cyclase, is involved in cGMP-PKG signaling pathway (Midttun et al., 2018). Furthermore, recent research finds that sildenafil increased cGMP by blocking PDE5 (phosphodiesterase-5) which can abolish the negative effect of hyperglycemia on SCs and exert the beneficial effects on DPN through the cGMP-PKG signaling pathway (Hendrix et al., 2013). It seems a reasonable deduction that overexpression of miR-212-5p may downregulate the level of Gucy1a3 in SCs contributing to DPN via cGMP-PKG signaling pathway. To further investigate the role of miR-212-5p in SCs of DPN, a subnetwork was extracted from the globe ceRNA network of DPN. The subnetwork showed that some lncRNAs such as LOC102550012, AABR07061328.1, AABR07063724.1, AABR07054488.1, and AABR07030791.1 may participate in the regulation of interaction of miR-212-5p and Gucy1a3. Among the mentioned lncRNAs, LOC102550012, which had the highest degree in the downregulated lncRNAs of ceRNA subnetwork and also had interaction with Gucy1a3, probably have the great possibility of competitively bounding with miR-212-5p, acting as the ceRNA and leading the expression level of Gucu1a3 changes. Above all, LOC102550012, miR-212-5p and Gucy1a3 might play an important role in the development of DPN related to dysfunction of SCs and need deeper studying and probing.

In summary, our study is the first to detect and analyze mRNAs, lncRNAs, and miRNAs changes in SCs from STZ-treated rats with DPN and reveal the novel interactions between differentially expressed RNAs and the pathogenesis of DPN. However, peripheral nerve system does not only consist of axons and SCs, mesenchymal cells, endothelial cell, immune cells like NK cells, and T cells are also abundant in nerves. Importantly, the interactions between them under the diabetic state still remain to be discussed. Moreover, the changes in protein expression level need further observation and exploration. Therefore, further studies are needed to find the complete proteomic and specific pathways of differentially expressed RNAs, the connections among different kinds of cells in peripheral nerve system during the process of DPN, and novel targets to treat DPN.

## Data Availability Statement

The RNA-seq data of Schwann cells from diabetic rats and control rats can be accessed online with the accession GEO: GSE142785 (https://www.ncbi.nlm.nih.gov/geo/query/acc.cgi?a cc=GSE142785).

## Ethics Statement

The animal study was reviewed and approved by the Animal Ethics Committee of Huazhong University of Science and Technology.

## Author Contributions

CW and YC designed the conceptual idea for this study. CW, XX, and JC performed the experiments and collected the data. YK and JG helped with data analysis. CW and HX performed the bioinformatic analysis. SR, TJ, and GG conducted the rats feed and DPN models. CW and XX wrote the manuscript. DD, XY, and H-GM revised the manuscript. MY and ZC provided study materials. All authors read and approved the final manuscript.

## Conflict of Interest

The authors declare that the research was conducted in the absence of any commercial or financial relationships that could be construed as a potential conflict of interest.

## Footnotes

1 http://www.geneontology.org

2 http://www.genome.jp/kegg/

3 https://cytoscape.org/
