# Supplementary material for: The Construction and Analysis of lncRNA–miRNA–mRNA Competing Endogenous RNA Network of Schwann Cells in Diabetic Peripheral Neuropathy
Source: Front Bioeng Biotechnol. 2020 May 25;8:490. doi: 10.3389/fbioe.2020.00490 (PMC7261901; doi:10.3389/fbioe.2020.00490)
Supplement: Supplementary file 1 [file Table_1.DOCX]

| Table S1. The primer sequences for qRT-PCR analysis | | |
| --- | --- | --- |
| Sez6l | Forward | TCATGGGCGAAGTGACCATCC |
|  | Reverse | GCTTCTGCTTCCAACGCATGT |
| Ocln | Forward | TCCAACGGCAAAGTGAATGGC |
|  | Reverse | CCACCTGTCGTGTAGTCGGTT |
| Pld4 | Forward | GGTGAAGGAGCTTGGCGCTAT |
|  | Reverse | AGGGAGGAGAGGCCGAGAAAT |
| Igdcc3 | Forward | GCACGGGGAACCTCATCATCT |
|  | Reverse | CTTGGCAGGTGAACATGGCTG |
| Esm1 | Forward | GGTGACGAGTTTGGTGTCTGT |
|  | Reverse | CTGCATCGCGCTCTGTTTGG |
| Slc7a9 | Forward | ATCTCCCCCAAGTCTGTGCTG |
|  | Reverse | GATGGCGAAGGATGAGGGCTT |
| Als2cl | Forward | ATGATGGCGAGTGGAGTCGAG |
|  | Reverse | ACCGGCCTGAAAATAGCCCTT |
| Gucy1a3 | Forward | GCCCCGATATTGCCTGTTTGG |
|  | Reverse | TGGAAGCTCCTCCCTCGATCT |
| Slco1a4 | Forward | GCTGCACACTTAGCATTCTGGC |
|  | Reverse | TAGTTGGTGCTGAACCCCTTCAT |
| Cxcl13 | Forward | CCTCCAGGCCACGGTATTCT |
|  | Reverse | GGCAGTAGGATTCACACATATAGC |
| Grem1 | Forward | CCACCTCCTGACAAGGCTCA |
|  | Reverse | CAGGGGCTGAGTTTTGCACC |
| Casq2 | Forward | GACAAGGTCGCACAGAAGCAG |
|  | Reverse | CCCCGTCAAACTCAATGGTGC |
| Tsga10ip | Forward | ATGCTACCTTGGCCTTCCTCG |
|  | Reverse | CTTGCGCCTCAGCTCCTCTAGT |
| AABR07045350.1 | Forward | CAAACCAGCACAAAGGAGAGG |
|  | Reverse | GTCTGTGTTGTCCTGAAGCTG |
| Lepr | Forward | GCTGCTCGGAACACTGTTAAT |
|  | Reverse | TGGACTGTTGGAAGGTTGGTA |
| Aox3 | Forward | GCCTCCCAATCTCTCCATGCT |
|  | Reverse | AGCTGGGCAGAGAAAGGGATG |
| AABR07061448.2 | Forward | ACCCATTGGACCAACAGTGTC |
|  | Reverse | TCATCCCCTTCACCTTTGAGA |
| AABR07053710.2 | Forward | GGTTGACTTGGCCATGATGTT |
|  | Reverse | TTGCAGGCTCCTTCTCATCTC |
| AABR07049695.2 | Forward | GCCACGGACAAGAACCTACTG |
|  | Reverse | AGTCAGCCGTCTGTCCAGTTC |
| AABR07051308.1 | Forward | AGAGCGTTTGTGTGGAAGGGA |
|  | Reverse | GGCAGCCCACAGAGATTACCA |
| rno-miR-155-3p | CCTCCTACCTGTTAGCATTA | |
| rno-miR-92a-3p | CTATTGCACTTGTCCCGGC | |
| rno-miR-205 | TTCATTCCACCGGAGTCTGT | |
| rno-miR-296-3p | AAAGGGTTGGGTGGAGGCT | |
| rno-miR-483-3p | AACTCCCCTCCCGTCTTGT | |
| rno-miR-212-5p | ACCTTGGCTCTAGACTGCTTA | |
| rno-miR-503-5p | TAGCAGCGGGAACAGTACTG | |
| β-actin | Forward | TCACTGTCCACCTTCCAGCAG |
|  | Reverse | ACGCAGCTCAGTAACAGTCCG |
| U6 | Forward | CCTGCTTCGGCAGCACA |
|  | Reverse | AACGCTTCACGAATTTGCGT |
