# Supplementary material for: The Construction and Analysis of lncRNA–miRNA–mRNA Competing Endogenous RNA Network of Schwann Cells in Diabetic Peripheral Neuropathy
Source: Front Bioeng Biotechnol. 2020 May 25;8:490. doi: 10.3389/fbioe.2020.00490 (PMC7261901; doi:10.3389/fbioe.2020.00490)
Supplement: Supplementary file 10 [file Table_10.DOCX]

Supplementary Material

# Supplementary data

**1.1 supplementary file 1** Total RNA Sample QC Report

# Supplementary Figures and Tables

## Supplementary Figures

**Supplementary Figure 1.** The whole lncRNA-mRNA co-expression network.

**Supplementary Figure 2.** The whole lncRNA-miRNA-mRNA ceRNA network.

**Supplementary Table 1.** Primers used for qRT-RCR in this study.

**Supplementary Table 2.** Differentially expressed mRNA transcripts from RNA-seq analysis. Information about the upregulated and downregulated mRNA transcripts in DPN rats compared with control rats.

**Supplementary Table 3.** Differentially expressed lncRNA transcripts from RNA-seq analysis. Information about the upregulated and downregulated lncRNA transcripts in DPN rats compared with control rats.

**Supplementary Table 4.** Differentially expressed miRNA transcripts from RNA-seq analysis. Information about the upregulated and downregulated miRNA transcripts in DPN rats compared with control rats.

**Supplementary Table 5.** GO analysis results.

**Supplementary Table 6.** Pathway analysis results.

**Supplementary Table 7.** Information about lncRNA-mRNA co-expression network.

**Supplementary Table 8.** Target gene prediction of differentially expressed miRNAs by miRanda and miRTarBase database.

**Supplementary Table 9.** Information about lncRNA-miRNA-mRNA ceRNA network.
