# Supplementary material for: The Construction and Analysis of lncRNA–miRNA–mRNA Competing Endogenous RNA Network of Schwann Cells in Diabetic Peripheral Neuropathy
Source: Front Bioeng Biotechnol. 2020 May 25;8:490. doi: 10.3389/fbioe.2020.00490 (PMC7261901; doi:10.3389/fbioe.2020.00490)
Supplement: Supplementary file 11 [file Data_Sheet_1.PDF]

其明信息
GMINIX

Total RNA Sample QC Report

1．Results

ID	Sample Name	Qubit concentration (ng/μl)	Total (μg)	Nano Drop A260/A280	4200 RIN	4200 28S/18S	Results
1	T2	924	41.58	2.10	10.0	2.6	A
2	T4	152.4	6.858	2.04	9.9	2.2	A
3	T7	1104	49.68	2.08	10.0	2.2	A
4	C5	342	15.39	2.10	10.0	2.3	A
5	C6	240	10.8	2.10	10.0	2.4	A
6	C10	195	8.775	2.08	10	2.3	A

2．Comprehensive assessment instructions

Level	Quantity	Quality	Problem	Suggestion
A	2 or more times	Acceptable	/	allows for subsequent experiments
B	1 time	Acceptable	/	allows for subsequent experiments / enable backup samples / resend samples
C	Less than 1 time	Acceptable	insufficient quantity	resend samples
D	/	not acceptable	partial degradation	resend samples
E	/	not acceptable	degradation	resend samples

3．4200 RIN

其明信息
GMINIX

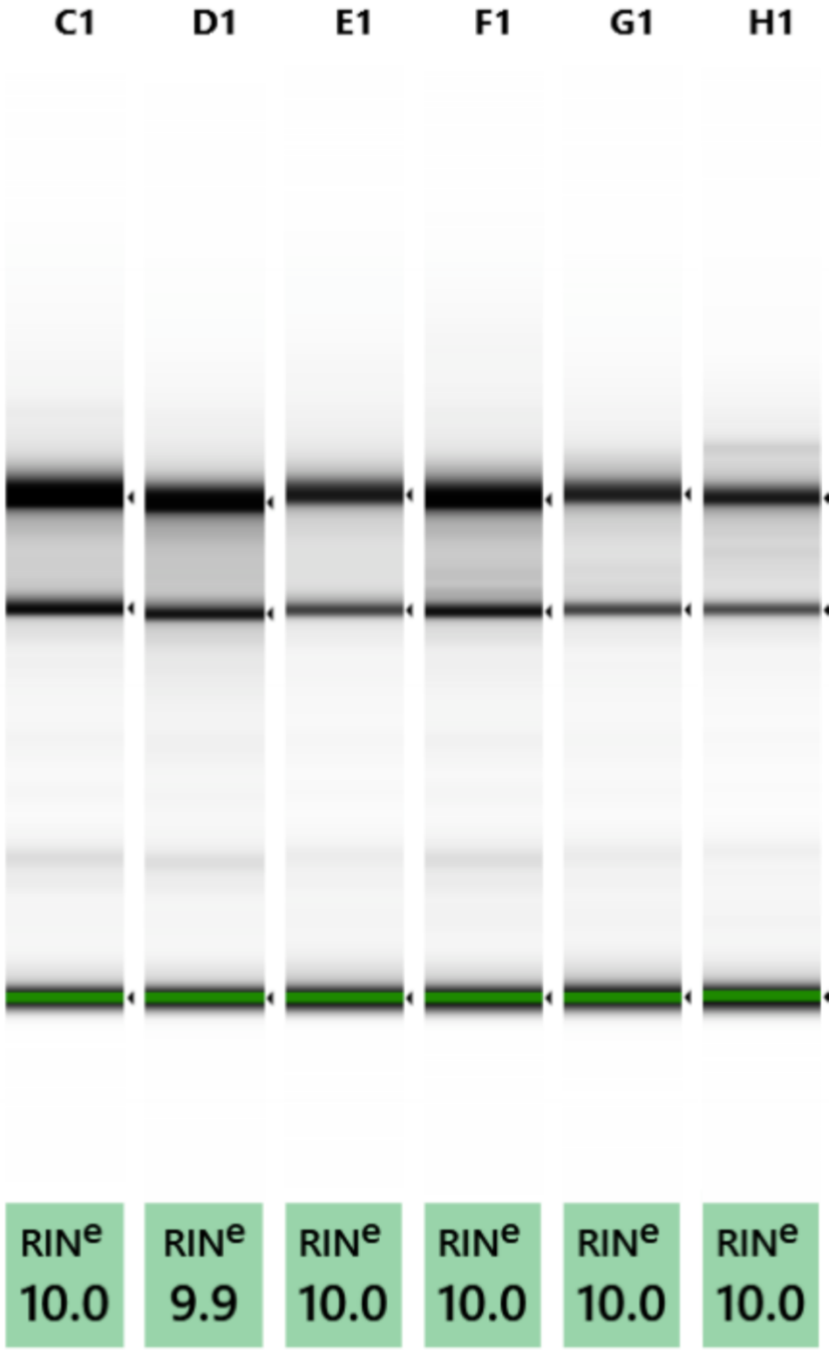

C1	10.0	2.6	103	T2
D1	9.9	2.2	92.3	T4
E1	10.0	2.2	56.1	T7
F1	10.0	2.3	86.3	C5
G1	10.0	2.4	51.8	C6
H1	10.0	2.3	41.6	C10

C1: T2

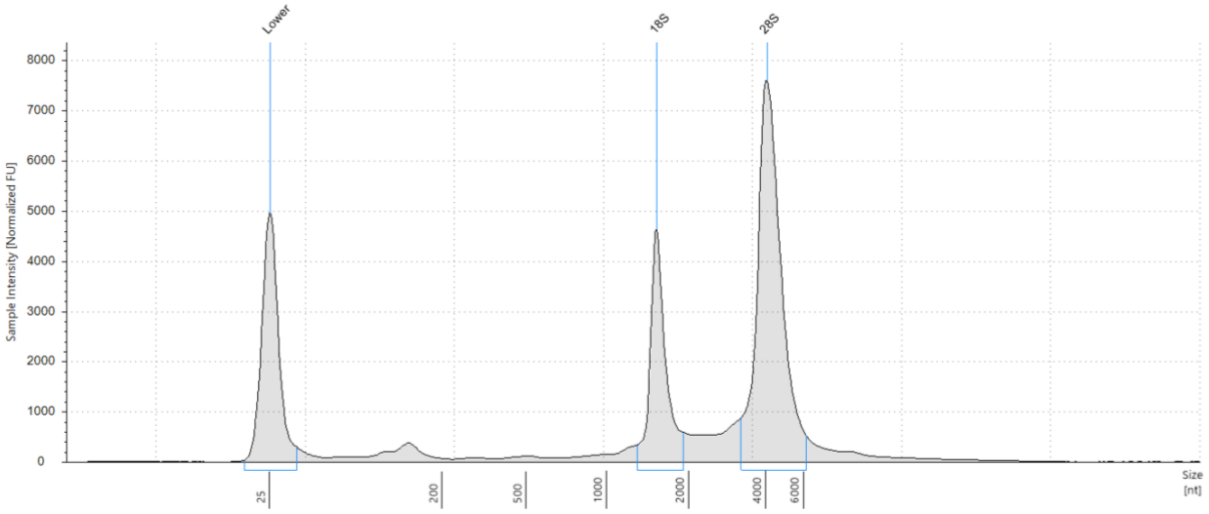

D1: T4

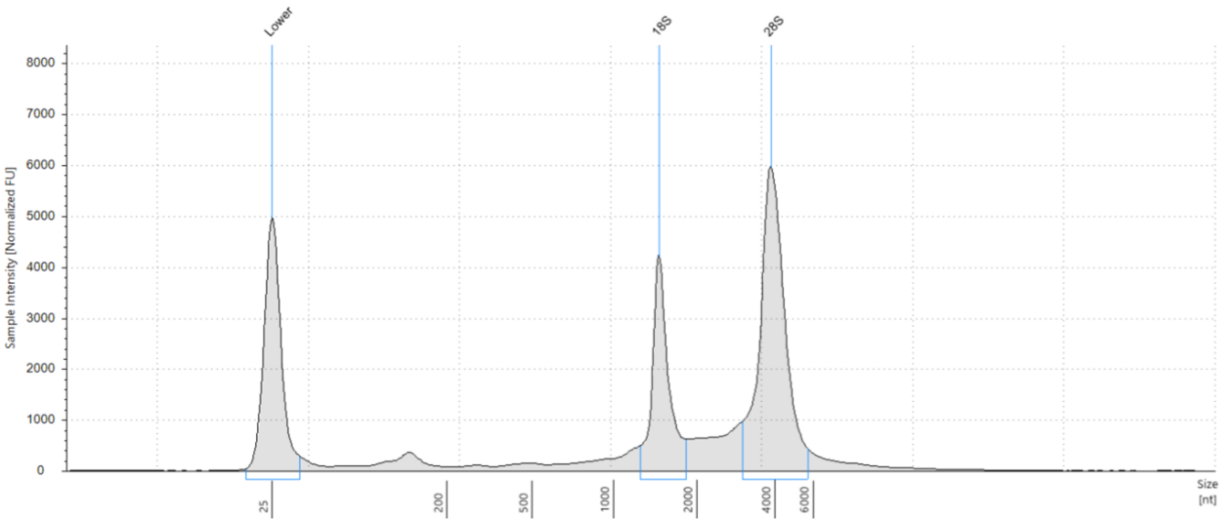

Sample Table

其明信息
GMINIX

E1: T7

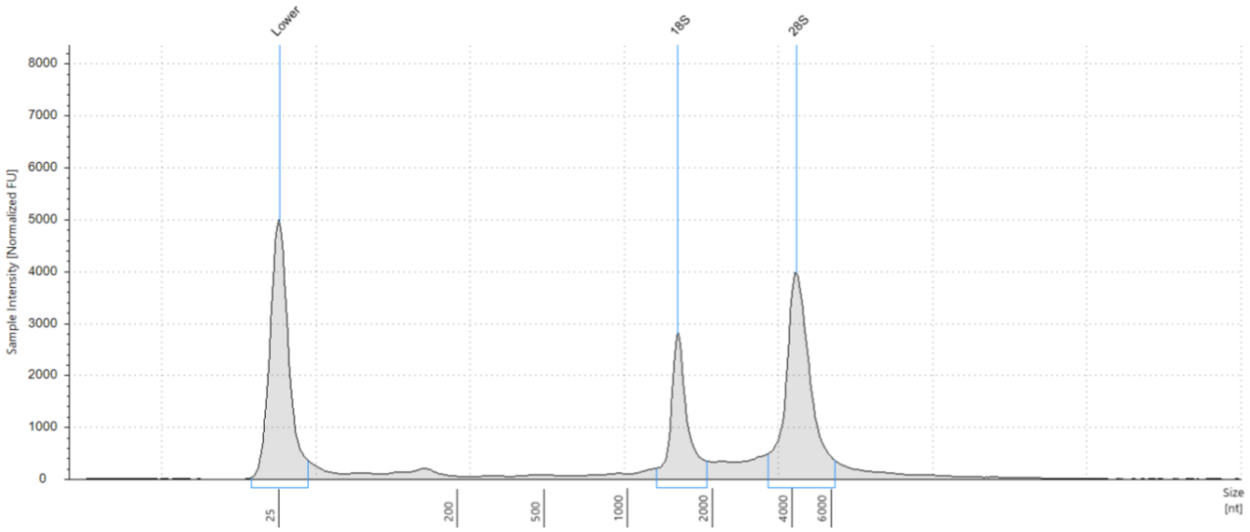

Sample Table

F1: C5

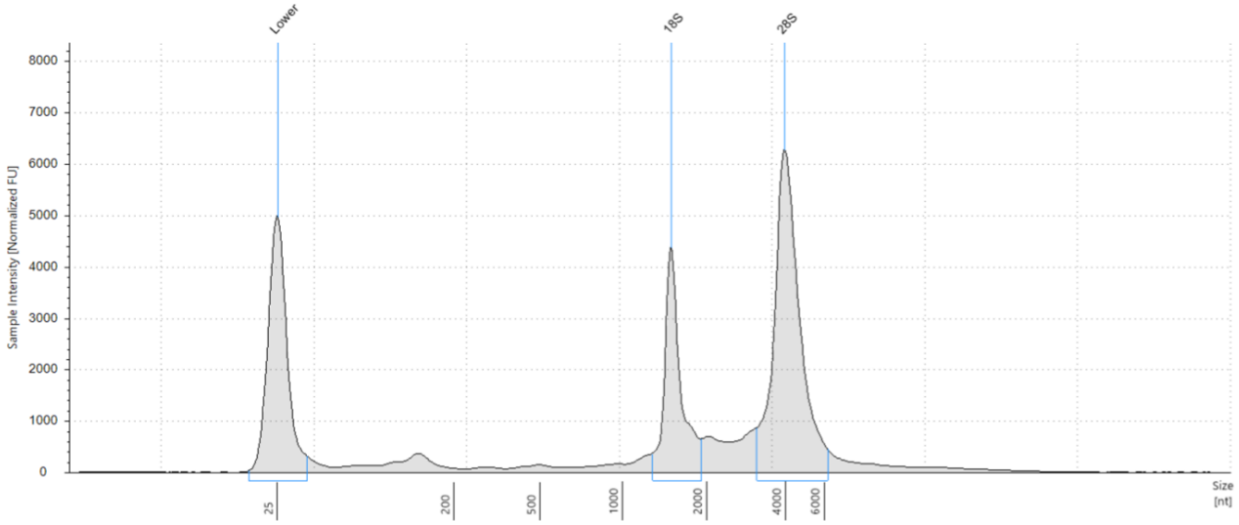

G1: C6

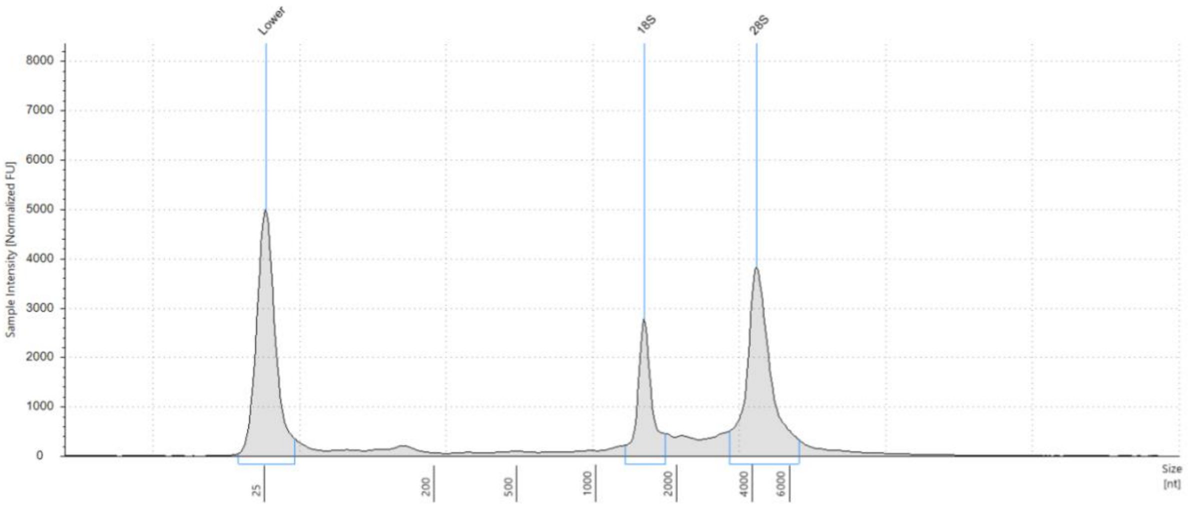
Lower
18S
28S
Sample Intensity [Normalized FU]
8000
7000
6000
5000
4000
3000
2000
1000
0
25
200
500
1000
2000
4000
6000
Size
[nt]

H1: C10

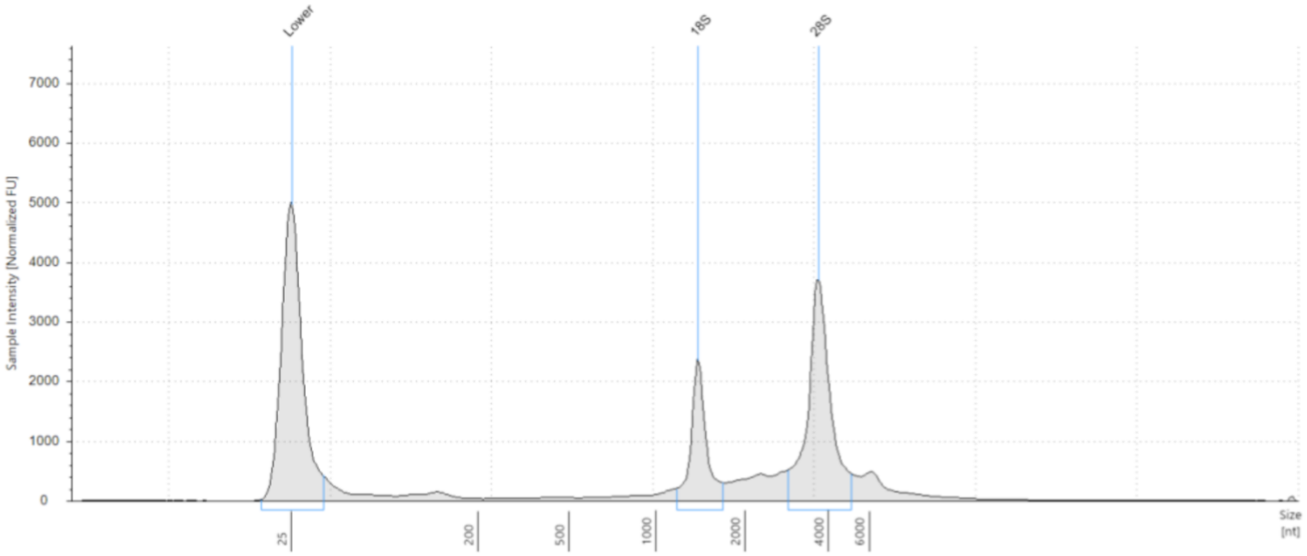
Lower
18S
28S
Sample Intensity [Normalized FU]
7000
6000
5000
4000
3000
2000
1000
0
25
200
500
1000
2000
4000
6000
Size
[nt]
