# Supplementary figures and images for: The Construction and Analysis of lncRNA–miRNA–mRNA Competing Endogenous RNA Network of Schwann Cells in Diabetic Peripheral Neuropathy
Source: Front Bioeng Biotechnol. 2020 May 25;8:490. doi: 10.3389/fbioe.2020.00490 (PMC7261901; doi:10.3389/fbioe.2020.00490)

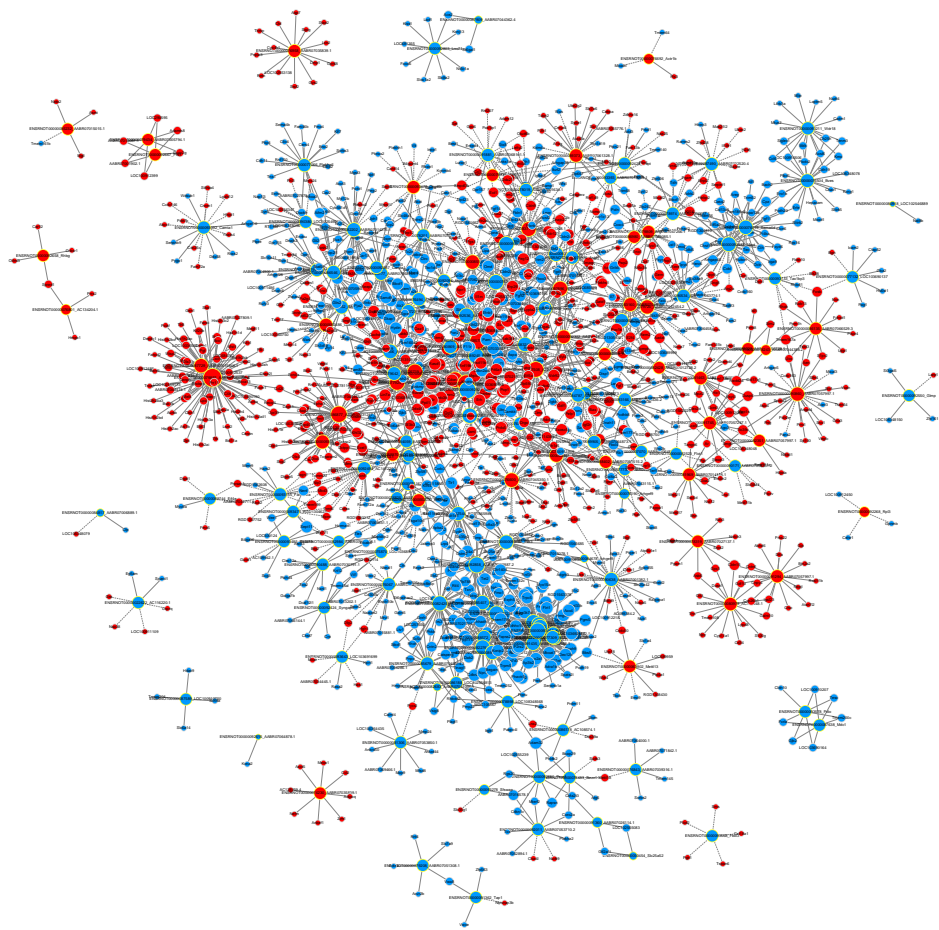

Supplement: Supplementary file 12 [file Image_1.PDF]

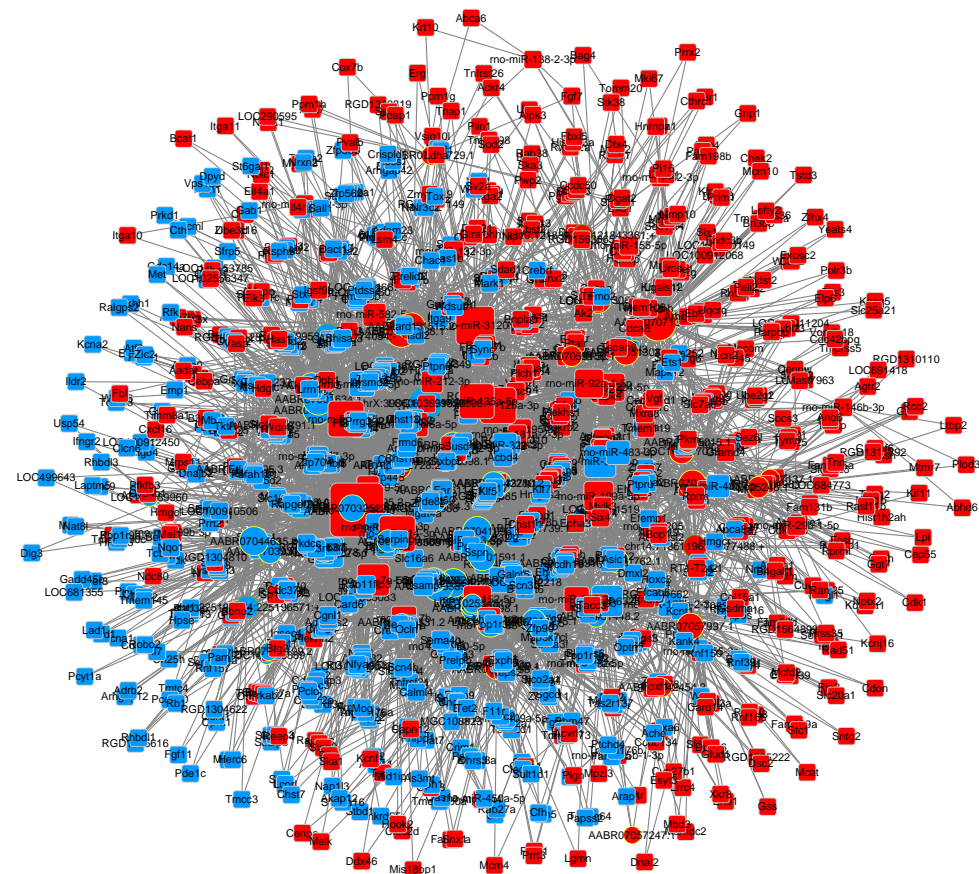

Supplement: Supplementary file 13 [file Image_2.PDF]
